# Causal Relationship Between Sjögren’s Syndrome and Atherosclerosis: A Bidirectional, Two-Sample, Mendelian Randomization Study

**DOI:** 10.7759/cureus.89571

**Published:** 2025-08-07

**Authors:** Luoyang Jing, Zhaolong Liu, Haolin Li, Xuemei Tian, Weiqing Li, Ping Chen, Haidong Wang

**Affiliations:** 1 School of Clinical Chinese Medicine, Gansu University of Chinese Medicine, Lanzhou, CHN; 2 Department of Rheumatology and Bone Diseases, Gansu Provincial Hospital of Traditional Chinese Medicine, Lanzhou, CHN

**Keywords:** cerebral atherosclerosis, coronary atherosclerosis, mendelian randomization, peripheral atherosclerosis, sjögren’s syndrome

## Abstract

Background: There is growing evidence that Sjögren’s syndrome (SS) and atherosclerosis (AS) might share underlying immunological and inflammatory processes. Observational data have pointed toward a potential association between SS and a heightened likelihood of developing AS, though the causal direction and specific dynamics of this relationship have not been clearly verified. This Mendelian randomization (MR) investigation opts to investigate potential bidirectional causality between SS and three types of AS: coronary, cerebral, and peripheral.

Methods: Genetic variants significantly linked to SS and multiple AS subtypes were obtained from large-scale genome-wide association studies (GWAS). The main approach used for causal estimation was the inverse-variance weighted (IVW) method. To ensure robustness, additional methods such as the weighted median, MR-Egger regression, simple mode, and weighted mode were also applied. To test result stability and detect pleiotropy, tools including Cochran’s Q test, MR-Egger intercept analysis, MR-Pleiotropy Residual Sum and Outlier (PRESSO) method, and leave-one-out analysis were performed.

Results: The results showed a notable genetic link between SS as well as an elevated coronary likelihood (OR = 1.046, 95%CI, 1.024-1.069;* P* = 5.52×10⁻⁵), cerebral (OR = 1.320, 95%CI, 1.040-1.677*; P* = 0.023), and peripheral (OR = 1.154, 95%CI, 1.082-1.231; *P* = 1.188×10⁻⁵) AS. Such findings imply that SS could independently elevate the risk for atherosclerotic disease. Conversely, reverse MR analysis suggested that peripheral AS could have a causal role in SS onset (OR = 1.751, 95%CI, 1.009-3.038; *P* = 0.046), whereas no significant reverse effect was observed from coronary or cerebral AS.

Conclusion: This study presents genetic evidence supporting a bidirectional causal link between SS and peripheral AS, and a unidirectional causal influence from SS toward both coronary and cerebral AS. The outcomes emphasize the importance of early cardiovascular screening and integrated management strategies addressing immune-vascular comorbidities in individuals diagnosed with SS.

## Introduction

Sjögren’s syndrome (SS) is a chronic immune-mediated disorder that predominantly targets the body’s exocrine glands, resulting in gradual functional decline [1]. A global analysis covering 21 epidemiological investigations reported an incidence of approximately 6.92 new cases per 100,000 individuals and a prevalence of 60.82 per 100,000. The condition demonstrates a marked gender disparity, affecting women approximately nine times more often than men [2]. Onset is most frequently observed between 40 and 60 years of age, with incidence increasing progressively with aging [3]. SS has an insidious onset and complex clinical presentation, mainly characterized by xerostomia and xerophthalmia due to progressive dysfunction of salivary and lacrimal glands. Individuals frequently experience rampant dental caries, recurrent parotid swelling, and joint pain. In more severe cases, the disease can impact several body systems, including the pulmonary and renal systems, nervous system, as well as heart and vessels, substantially impairing quality of life and even threatening survival [4]. Unfortunately, due to the heterogeneity and diagnostic challenges of SS, it is often underdiagnosed, and systemic involvement may already be present at the time of diagnosis[5].

Atherosclerosis (AS) is a long-standing arterial condition driven by lipid build-up in the innermost layer of the arteries, leading to plaque development, reduced arterial elasticity, and narrowing of the lumen. This disease process is closely associated with persistent low-grade inflammation [6]. Depending on the anatomical site affected, AS is classified into coronary, cerebral, and peripheral subtypes, linked to conditions like coronary artery illness, stroke from ischemia, and peripheral artery disease, respectively [7]. AS ranks one of the leading contributors to mortality associated with vascular conditions and involves the engagement of adaptive as well as innate immune mechanisms [8]. As stated by the WHO, cardiovascular conditions are responsible for an estimated 17.9 million deaths per year, constituting nearly 32% of global mortality [9].

In recent times, cardiovascular conditions have been increasingly acknowledged as a primary contributor to mortality in those with SS. In comparison to individuals without SS, patients with SS face significantly greater risks for various cardiovascular issues like coronary artery disease, stroke from ischemia, cardiac failure, as well as venous thromboembolism, with respective increases in risk of 34%, 46%, 154%, and 78% [10]. Recent studies also reveal that individuals with SS often show heightened signs of hidden atherosclerosis, like thicker carotid artery walls and elevated arterial rigidity, even without typical cardiovascular risk contributors [11]. Furthermore, prolonged disease duration in SS is commonly associated with more advanced coronary calcification and myocardial tissue fibrosis. Case reports have described patients with SS developing recurrent cerebral infarctions, progressive cerebral artery stenosis, and multiple brain infarcts, even in the absence of conventional risk factors, suggesting an underlying immune-mediated vasculopathy [12]. Endothelial dysfunction and localized vascular inflammation, key early pathological events in AS, have also been frequently observed in SS patients [13]. Notwithstanding these findings, the causality of the SS-AS relationship is uncertain owing to confounding and reverse causality in observation designs.

Mendelian randomization (MR) is a genetic approach utilizing inherited versions as instrumental variables for testing for cause-and-effect relations between exposures and disease outcomes. This method is especially useful in avoiding the effects of confounding variables and bias due to the possibility of reverse causation, typical problems of conventional observation studies. To address these limitations, MR has emerged as a powerful method that uses genetic variants as instrumental variables to infer causal relationships between exposures and outcomes. Because genetic variants are randomly assigned at conception, MR can effectively minimize confounding and reverse causality, thus offering greater credibility than conventional observational designs.MR is now an established, popular method in investigating the etiology and causal pathways of diseases [14]. This study utilized a bidirectional two-sample MR approach to explore if SS and AS share a direct causal link, with the objective of providing genetic insights useful for the eventual early diagnosis, prevention, and management of cardiovascular complications in SS patients.

## Materials and methods

Study design

**Figure 1:**
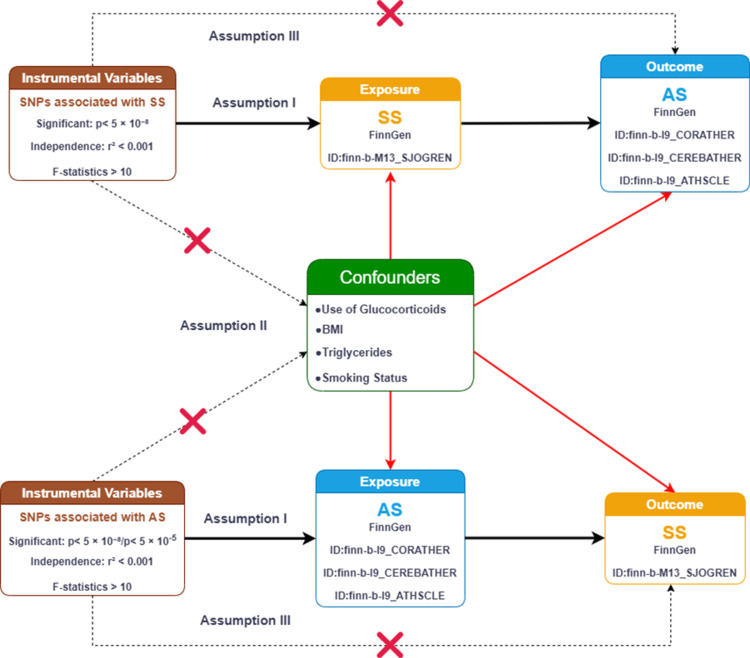
The work flow of this study SS: Sjögren’s syndrome; AS: atherosclerosis

A two-sample bidirectional MR approach was employed to investigate potential causal interactions within SS and three different types of AS, which include coronary, cerebral, and peripheral AS. In order to test for inconsistency in the instrumental variables (IVs), Cochran’s Q statistic was utilized. Various sensitivity checks were conducted in order to ensure that the causal estimates are robust as well as consistent. For this MR analysis to provide valid conclusions, it was based on three necessary assumptions: (i) Relevance: the single nucleotide polymorphisms (SNPs) serving as tools need to correlate with the exposure variable, (ii) Independence: the IVs should remain independent of outside confounding influences that simultaneously affect both exposure factors as well as results, and (iii) Exclusion Restriction: the pathway from instrumental variables should influence restult solely via their link to exposure pathway, excluding alternative indirect effects [15]. An illustration outlining the overall study framework is presented in Figure 1.

Data sources

Information on both exposures and outcomes was sourced using genome-wide association studies (GWAS) data made openly accessible by the FinnGen [16]. A summary of the datasets used can be found in Table 1.

**Table 1:** Information on data related to atherosclerosis (AS) and Sjögren’s syndrome (SS) GWAS: genome-wide association studies

Data	Population	Case (n)	Control (n)	Sample size(n)	SNP	GWAS ID
Coronary atherosclerosis	European	63307	416171	479478	21306500	finn-b-I9_CORATHER
Cerebral atherosclerosis	European	440	499908	500348	21306789	finn-b-I9_CEREBATHER
Peripheral atherosclerosis	European	19807	463106	482913	21306543	finn-b-I9_ATHSCLE
SS	European	3309	484260	487569	21319120	finn-b-M13_SJOGREN

Instrumental variable selection

SNPs showing strong associations with SS meeting the genome-wide significance criterion (P < 5 x 10⁻⁸) were selected as candidate IVs. To control for linkage disequilibrium (LD) and ensure independence among variants, SNPs were filtered based on a minimum physical distance of over 10,000 kilobases and an LD criterion of r² < 0.001. In cases where the number of qualified SNPs was insufficient, a relaxed threshold of P < 5 × 10⁻⁵ was applied to broaden inclusion [17]. Weak instruments were excluded using the F-statistic, with only those having F > 10 retained to ensure instrument strength. The F-statistic was calculated using the formula: $F = \frac{N - K - 1}{K} \times \frac{R^{2}}{1 - R^{2}}$ where N is the sample size, K is the number of instrumental variables (IVs), and R² represents the proportion of variance in the exposure explained by the IVs​​​​​​​ [18]. A higher F value indicates a stronger instrument, with values above 10 commonly considered sufficient to reduce weak instrument bias.

MR analysis

In investigating causal associations among SS and each subtype of AS, the following approaches were applied: IVW, MR-Egger, weighted median, simple mode, and weighted mode. IVW was used as the primary investigation method due to its property of yielding consistent estimates provided that the IVs meet validity assumptions. Causal effects were reported as ORs with 95% CIs.

Testing for heterogeneity and pleiotropy

Cochran’s Q test was employed to assess instrumental variable heterogeneity, with heterogeneity regarded as significant when P< 0.05. For assessing the possibility of directional horizontal pleiotropy, in which the instrumental variables would impact the effect through non-causal channels, the MR-Egger intercept was evaluated.

Sensitivity analysis

For consistency checks, leave-one-out testing was applied, excluding one SNP at a time in order to see if any single SNP disproportionately impacted overall MR estimates, thus testing for the robustness of the results.

Reverse MR analysis

An additional reverse MR analysis was conducted, with the three AS subtypes as exposures and SS as the disease state. The pipeline of MR was the same, utilizing the same quality controls for ensuring consistency and reliability between analyses.

Data analysis

All calculations were completed using R software v4.4.2 (R Foundation for Statistical Computing, Vienna, Austria), supplemented with the TwoSampleMR package (v0.6.8) (https://mrcieu.github.io/TwoSampleMR/news/index.html). IVW was the primary method for assessing causality. Both the MR-Egger and weighted median regression methods were applied to assess the robustness of the estimates. p-values, ORs, and 95% CI were used to present the findings.

## Results

Instrumental variables

A total of eight SNPs were found that fulfilled the selection criteria as appropriate instruments for SS. Each of these SNPs had an F-statistic exceeding 10, suggesting strong instrument power with less risk of weak instrument bias. Each of these variants also fulfilled the important assumptions necessary for valid MR, increasing the credibility of the causal interpretations derived from the analysis.

MR analysis

**Figure 2:**
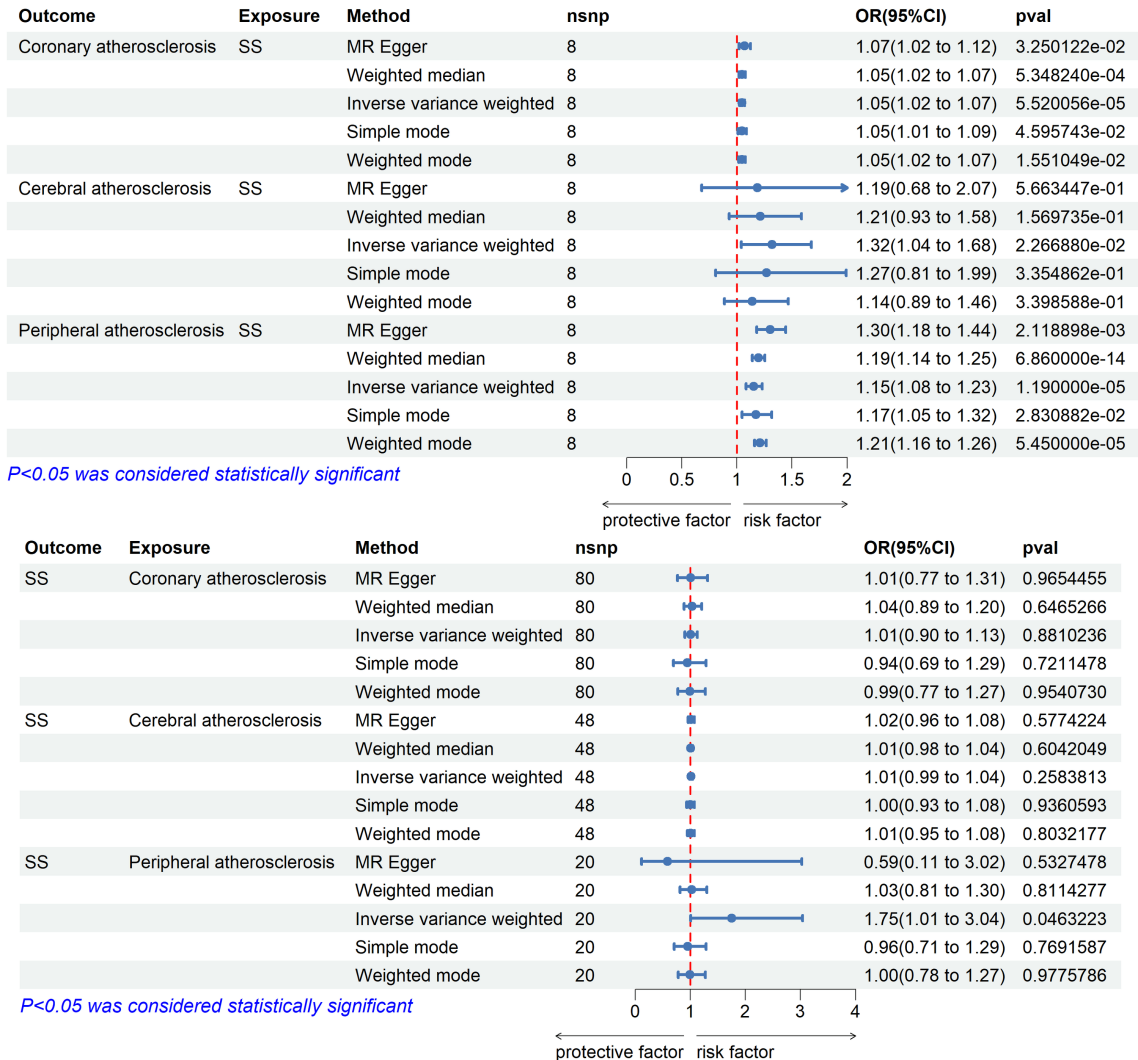
Forest plot of two-sample Mendelian randomization analysis

**Figure 3:**
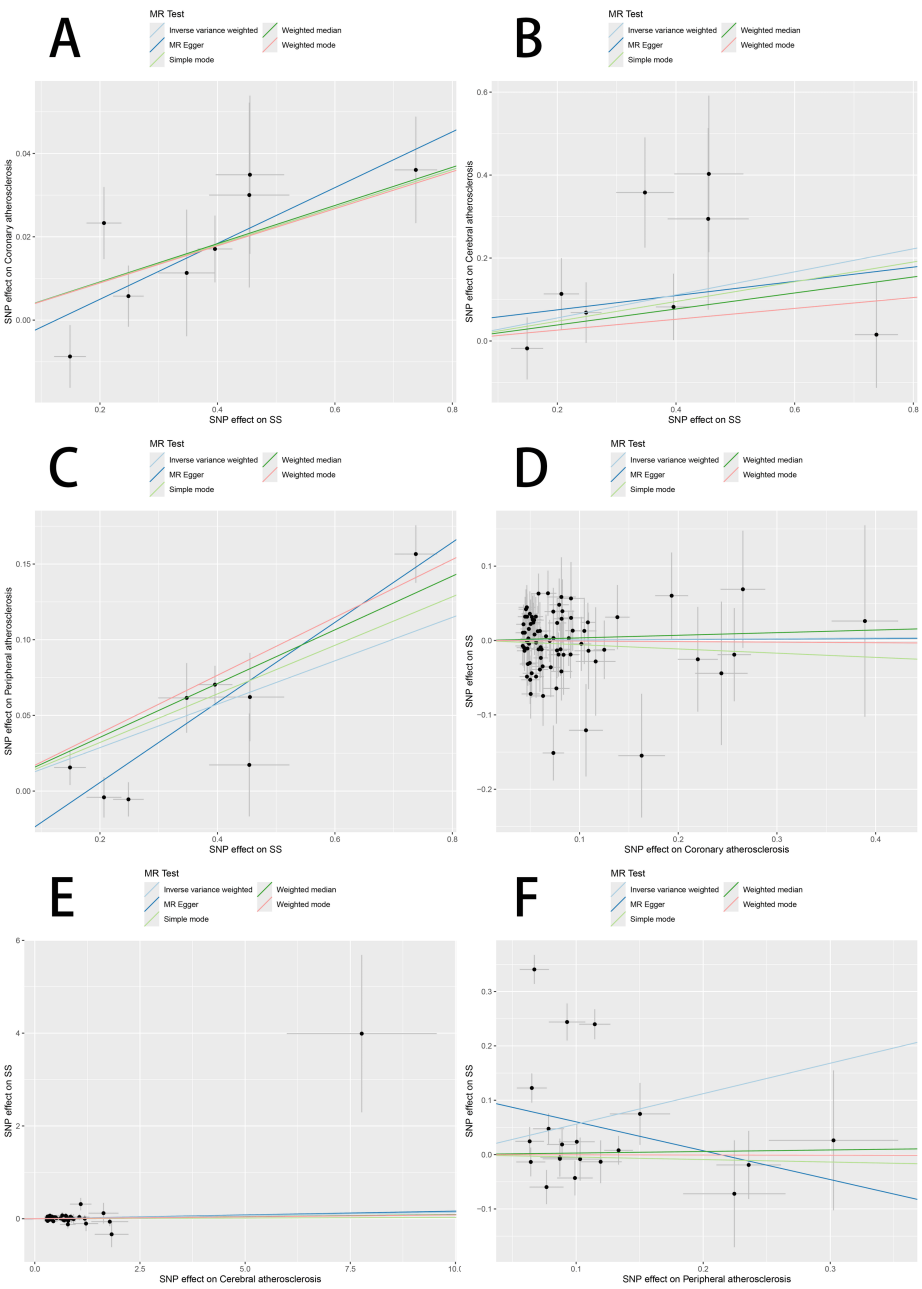
Scatter plot of two-sample Mendelian randomization analysis MR: Mendelian randomization; SNP: single nucleotide polymorphism; SS: Sjögren’s syndrome

MR findings indicated statistically significant causal associations among SS as well as each of the three forms of AS. For coronary AS, IVW generated an OR of 1.046 (95%CI, 1.024-1.069; P = 5.52 × 10⁻⁵). For cerebral AS, OR was 1.320 (95%CI, 1.040-1.677; P = 0.023), whereas for peripheral AS, the OR was 1.154 (95%CI: 1.082-1.231; P = 1.188 × 10⁻⁵). These findings lend support for SS as a possible genetic contributor to each of the three AS forms. Visual representations in the form of forest and scatter plots of the MR estimates are provided in Figures 2, 3.

Heterogeneity and horizontal pleiotropy

**Figure 4:**
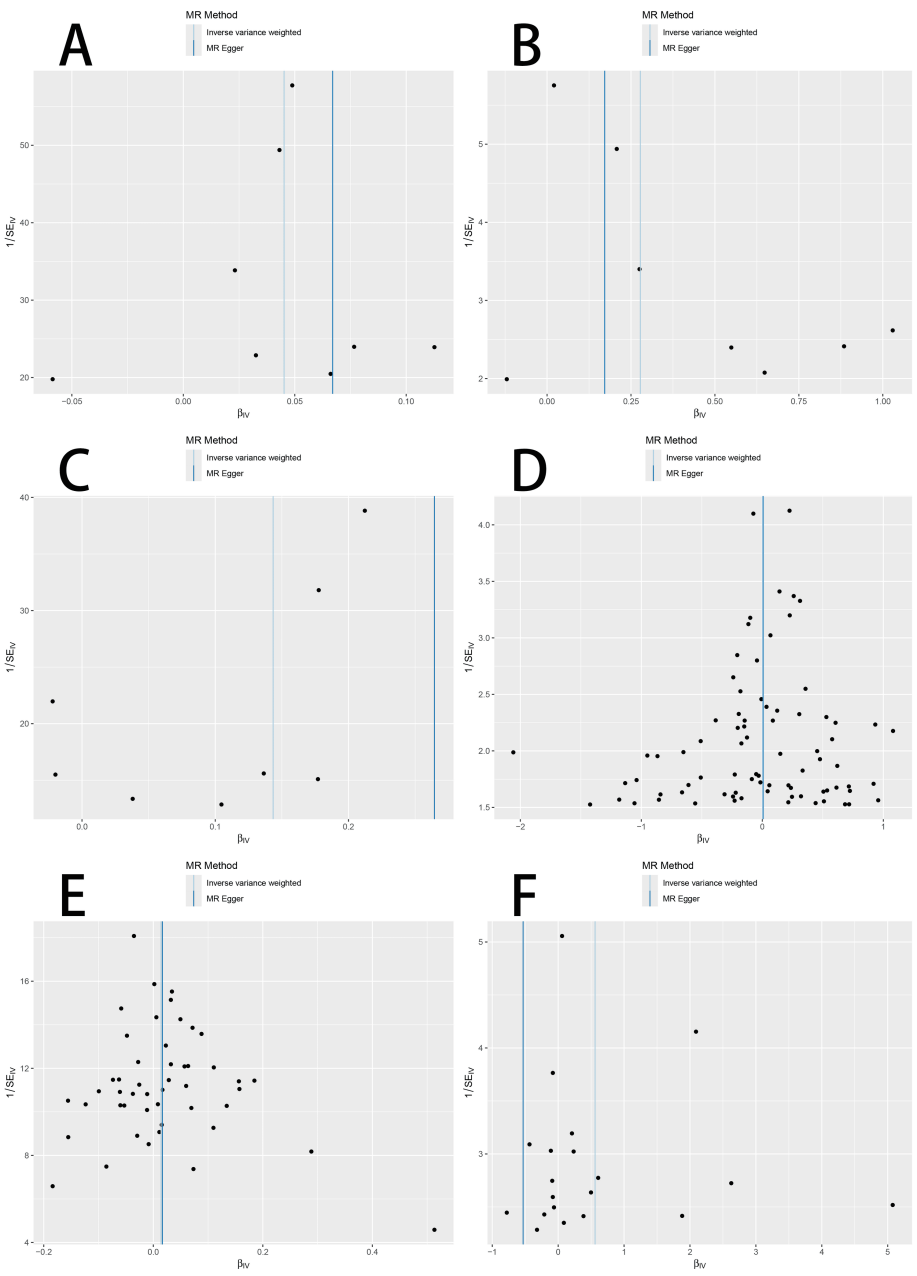
Funnel plot of two-sample Mendelian randomization analysis MR: Mendelian randomization

Cochran’s Q test indicated an absence of meaningful variation within the instrumental variables for coronary (P = 0.309) and cerebral AS (P = 0.191). However, peripheral AS showed significant heterogeneity (P = 0.030). Visual representations using funnel plots are provided in Figure 4.

The MR-Egger intercept analysis revealed no signs of directional pleiotropy for coronary (intercept = -0.0084, P = 0.349) or cerebral AS (intercept = 0.0407, P = 0.688). Conversely, the results indicated evidence of horizontal pleiotropy in the peripheral AS analysis (intercept = -0.0472, P = 0.037). To address this, MR-PRESSO was employed to detect and remove SNP outliers. A full summary of these findings can be found in Tables 2, 3.

**Table 2:** Results of the Cochran’s Q test MR: Mendelian randomization; SS: Sjögren’s syndrome; IVW: inverse-variance weighted

Outcome	Exposure	Method	Q	p value
Coronary atherosclerosis	SS	MR Egger	7.060	0.315
		IVW	8.272	0.309
Cerebral atherosclerosis	SS	MR Egger	9.672	0.139
		IVW	9.959	0.191
Peripheral atherosclerosis	SS	MR Egger	13.955	0.030
		IVW	30.453	7.840
SS	Coronary atherosclerosis	MR Egger	92.350	0.128
		IVW	92.351	0.145
SS	Cerebral atherosclerosis	MR Egger	45.003	0.514
		IVW	45.011	0.555
SS	Peripheral atherosclerosis	MR Egger	242.064	3.34e-41
		IVW	267.820	7.47e-46

**Table 3:** Results of the Mendelian randomization-Egger intercept test SS: Sjögren’s syndrome

Outcome	Exposure	Egger_intercept	p value
Coronary atherosclerosis	SS	-0.0084	0.349
Cerebral atherosclerosis	SS	0.0407	0.688
Peripheral atherosclerosis	SS	-0.0472	0.037
SS	Coronary atherosclerosis	0.0002	0.984
SS	Cerebral atherosclerosis	-0.0012	0.930
SS	Peripheral atherosclerosis	0.1133	0.183

Sensitivity analysis

**Figure 5:**
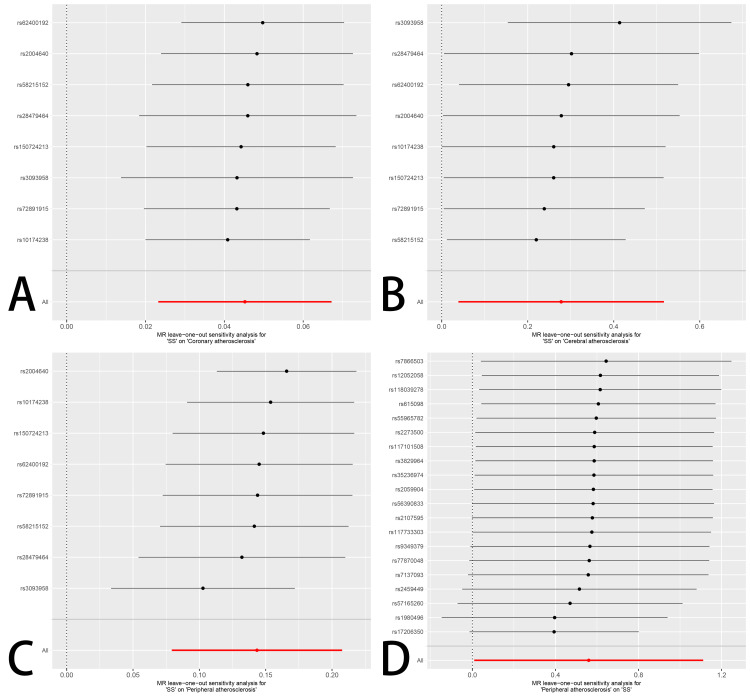
Results of Mendelian randomization leave-one-out sensitivity analysis MR: Mendelian randomization; SS: Sjögren’s syndrome

The leave-one-out analysis, in which each SNP was excluded one at a time, demonstrated that the remaining estimates consistently stayed above the null threshold, indicating strong consistency across variants. This affirmed the robustness and reliability of the MR findings. The corresponding plots can be found in Figure 5.

Reverse-direction MR analysis

When evaluating causality in the reverse direction, treating AS subtypes as exposures and SS as the outcome, only peripheral AS demonstrated a significant causal relationship. Twenty SNPs were selected as tools for instrumental variable analysis. Applying the IVW approach, the estimated OR was 1.751 (95%CI, 1.009-3.038; P < 0.05), suggesting a possible causal effect. Considerable variation among instruments was revealed using Cochran’s Q analysis (P = 7.47 × 10⁻⁴⁶), though the MR-Egger intercept (-0.000195, P = 0.984) provided no indication of directional pleiotropy. Leave-one-out testing confirmed the reliability of this finding. Supporting data and visual summaries are illustrated in Figures 2, 4, 5.

## Discussion

This study, employing a bidirectional two-sample MR strategy, represents the first comprehensive attempt to assess the causal connection between SS and the three principal subtypes of AS: coronary, cerebral, and peripheral. The analysis demonstrated that genetic predisposition to SS is significantly associated with heightened risks of all three AS forms, implying that SS may act as an independent etiological factor in atherosclerotic disease. Additionally, reverse MR analysis revealed a potential causal influence of peripheral AS on SS, suggesting the existence of a possible bidirectional disease mechanism.

SS is a chronic autoimmune disease targeting exocrine glands in the majority of cases, but affecting multiple organ systems in some instances as well. It has increasingly been linked with accelerated vascular injury and increased risk of early-onset cardiovascular disease [19]. In observational studies, repeatedly higher rates of myocardial infarction, stroke, and heart failure have been observed among individuals with SS [20]. However, such studies are prone to confounding variables and reverse causality, making it impossible to draw certain conclusions about causality. By utilizing genetic proxies for SS in this MR analysis, the evidence for SS making a causal contribution towards AS development is reinforced.

The relationship between SS and the development of AS most probably stems from ongoing immune system stimulation and inflammation. SS is characterized by high levels of inflammation-associated molecules like C-reactive protein (CRP), interleukin-6 (IL-6), tumor necrosis factor-α (TNF-α), and interferon-γ (IFN-γ), triggering the activation of cytokines such as endothelial and smooth muscle cells in vascular tissue, leading to the expression of adhesion molecules like VCAM-1 and ICAM-1, as well as numerous chemokines that support cellular infiltration into the artery wall as well as inflammation in the artery wall [21]. This inflammatory environment allows for the entry of T cells as well as monocytes into the wall of the artery, advancing the development as well as progression of atherosclerotic plaques. Moreover, SS patients regularly exhibit dysfunction of the endothelium, carotid intima-media thickening (IMT), as well as enhanced arterial stiffness-indicators of earlier, asymptomatic atherosclerosis[22,23].

Further evidence highlights that overactivation of neutrophils in SS can lead to the generation of neutrophil extracellular traps (NETs), which harm endothelial cells directly and activate plasmacytoid dendritic cells (pDCs). This activation promotes the secretion of type I interferons (IFN-I), amplifying the inflammatory cascade, attracting more immune cells, and worsening vascular injury, all of which contribute to plaque formation and vulnerability [24]. Altogether, these findings underscore the need to regard SS not only as an autoimmune disorder but also as a systemic condition with significant vascular implications-an “immune-vascular comorbidity” with elevated cardiovascular risk.

In the reverse MR analysis, only peripheral AS exhibited a statistically significant causal effect on SS. Although sensitivity analyses did not reveal evidence of directional pleiotropy, this result should be interpreted with caution. The possibility of horizontal pleiotropy or residual confounding due to imperfect SNP harmonization cannot be entirely excluded. Despite these limitations, the observed association is supported by plausible pathophysiological mechanisms. Peripheral arterial disease is often accompanied by chronic ischemia, hypoperfusion, and metabolic stress, which may trigger the release of immunogenic antigens and activate innate and adaptive immune responses [25]. Moreover, the inflammatory environment of atherosclerosis, enriched with interleukin-1 beta (IL-1β) and oxidative stress products, can promote autoantigen exposure and neo-epitope formation. In genetically susceptible individuals, this may contribute to the breakdown of immune tolerance and activation of autoreactive T and B cells​​​​​​​ [26]. Thus, the immune system may act not only as a target but also as a participant in the pathogenesis of As, potentially initiating or exacerbating autoimmune diseases such as SS.

From a clinical perspective, cardiovascular risk assessment should be integrated into the routine management of SS patients, with special attention to peripheral arterial function. Non-invasive measures such as the ankle-brachial index (ABI) and pulse wave velocity (PWV) may serve as useful tools for early vascular monitoring [27]. Moreover, considering the shared inflammatory pathways, such as IFN-I signaling, IL-6, and endothelial dysfunction, between SS and AS, targeted immunotherapy may not only control systemic inflammation and glandular damage but also delay atherosclerotic progression, offering a more comprehensive therapeutic strategy for patients with systemic autoimmune diseases [13,28].

Nevertheless, this study has several limitations. First, the GWAS summary statistics used were primarily derived from individuals of European ancestry, which may limit the generalizability of the findings to other populations. Second, the definition of SS did not distinguish between primary and secondary subtypes, which may introduce phenotypic heterogeneity. Third, although MR can reduce confounding and reverse causality, it cannot fully eliminate the potential influence of horizontal pleiotropy or genetic linkage.

In future research, the use of large-scale biobank resources (e.g., UK Biobank) and the establishment of prospective SS registries would be valuable for improving phenotypic accuracy and follow-up validation. Multi-ethnic cohorts with larger sample sizes, combined with multi-omics data (e.g., transcriptomics, proteomics, metabolomics), and detailed clinical phenotyping (e.g., European Alliance of Associations for Rheumatology Sjögren's Syndrome Disease Activity Index (ESSDAI) scores, autoantibody profiles) should be integrated. These efforts would help construct a more precise immuno-AS model and support early screening, risk stratification, and targeted intervention strategies in autoimmune disease-associated AS.

## Conclusions

Utilizing a two-sample MR test implemented in both directions, the investigation thoroughly investigated to determine if a cause-and-effect relationship is present involving SS and three key types of AS: coronary, cerebral, and peripheral. The findings offered genetic support for a significant causal role of SS in increasing susceptibility to all examined AS subtypes, indicating that SS may independently elevate the risk for atherosclerotic disease. Additionally, reverse MR analysis suggested that peripheral AS could also influence the development of SS, pointing to a potentially bidirectional causal connection between these conditions.

These findings offer novel insights into the immuno-cardiovascular connection and emphasize the need for early identification and integrated cardiovascular risk assessment in patients with SS. Future research should incorporate multi-ethnic populations and multi-omics data to further elucidate underlying mechanisms and guide individualized prevention and treatment strategies for autoimmune disease-related AS.
